# Evaluation of the Stomatognathic System before and after Osteopathic Manipulative Treatment in 120 Healthy People by Using Surface Electromyography

**DOI:** 10.3390/ijerph17093250

**Published:** 2020-05-07

**Authors:** Andrea Manzotti, Chiara Viganoni, Dorina Lauritano, Silvia Bernasconi, Alice Paparo, Rachele Risso, Alessandro Nanussi

**Affiliations:** 1 SOMA–osteopathic Institute of Milan, 20126 Milan, Italy; andreamanzotti@soma-osteopatia.it (A.M.); bernasconi.s@hotmail.it (S.B.); alice.paparo9@gmail.com (A.P.); super-ginger@hotmail.it (R.R.); nanussi@tiscalinet.it (A.N.); 2 Department of Medicine and Surgery, Centre of Neuroscience of Milan, University of Milano-Bicocca, 20126 Milan, Italy; c.viganoni@campus.unimib.it

**Keywords:** osteopathic manipulative treatment, masticatory muscles, electromyography, occlusion, temporomandibular joint

## Abstract

*Objective:* To investigate the action of osteopathic manipulative treatment on the muscular activity of the stomatognathic apparatus by using surface electromyography (sEMG). *Material and Methods:* Surface electromyography (sEMG) was performed on the masseter and anterior temporalis muscles of 120 subjects (73 F; 47 M), both at time T0 and T2. The sample was divided into three randomized groups of 40 subjects each: control, placebo, and osteopathic manipulative treatment (OMT). In the T1 interval between the two evaluations, the control group was not treated, the placebo group underwent a placebo treatment, and the OMT group underwent manipulative treatment. The mean value of each measurement and its coefficient of variation, between time T0 and T2, were calculated for both the intragroup (OMT, placebo, control) and the intergroup (OMT-placebo, OMT-control). *Outcomes:* In 40% of the subjects, statistically significant improvements were highlighted in the OMT. Whereas, the statistically significant results of the placebo and control groups were 7.5% and 17.5%, respectively, of which more than 75% moved away from the physiological range, showing a worsening of the muscular activity. This analysis showed statistically significant variations (*p* ≤ 0.05) in the OMT group compared to the placebo and the control groups. *Conclusions:* OMT determines variations of the activity of masticatory muscles.

## 1. Introduction

The relationship between dental occlusion, body posture, and temporomandibular disorders (TMD) is a controversial topic in dentistry. At present, literature data are mostly based on the effects of dental occlusion on head and body posture, but limited information is available on the inverse effects of posture on dental occlusion [1].

What is known is that TMDs have a multifactorial etiology that includes a broad range of phenotypic risk factors, as reported in the Orofacial Pain Prospective Evaluation and Risk Assessment Study (OPPERA) [2], and that it is a complex disorder often characterized by the co-occurrence of multiple conditions. Hence, it is no longer appropriate to consider it solely as a localized orofacial pain condition as the majority of people with chronic TMDs present a multisystem disorder with overlapping co-morbidity [3]. For this reason, the authors consider an interdisciplinary approach essential for the treatment of TMDs.

Furthermore, TMDs are the second most common musculoskeletal condition after chronic low back pain, as reported by the National Institute of Dental and Craniofacial Research. Thus, the need for first-line conservative treatment like osteopathic manipulative treatment (OMT) is recognized [4].

Osteopathic medicine is a noninvasive, drug-free manual medicine, classified as a complementary and alternative medicine. Its goal is the care of the individual in its entirety and works through manual manipulation techniques [5]. The OMT is a hands-on approach that has the objective of improving structures that inhibit the body’s function or homeostasis [6].

Alterations in the masticatory muscles, neck muscles, and occlusion can be considered causal factors of imbalance of the postural muscular chains [7]. Therefore, therapies that seek occlusal restoration, such as muscle relaxation techniques, should lead to an improvement in the overall equilibrium of the neuromuscular system and in the posture [8]. Furthermore, recent studies have shown that trigeminal afferents and alterations in the stomatognathic system are related to proprioception, visual stabilization, and postural stability [9,10].

The objective of this study was to investigate the action of osteopathic manipulative treatment on the muscular activity of the stomatognathic apparatus and on mandibular posture compensations by means of surface electromyography (sEMG).

Although the use of this device as a stand-alone diagnostic tool has raised strong negative criticism because of the absence of normative values [11], its role in a controlled research setting is recognized to have scientific merit [12].

Furthermore, the focus of this research was not to diagnose nor to treat TMDs, but to validate the effectiveness of OMT in terms of the change in muscular patterns when testing healthy subjects, so the choice of this instrument is related to its noninvasiveness.

## 2. Materials and Methods

The study was a randomized double-blind controlled trial (RCT) and the sample was made up of 120 healthy adult subjects (47 males and 73 females) between 19 and 62 years old (the average sample age was: 25.64 years), who all voluntarily participated in the study. As a result of the lack of research in this field, we did not conduct a power analysis to determine the sample size needed for this study. We decided to involve 120 patients considering this number sufficient for the representativeness of the sample for the validation of OMT. The power of the study was calculated retrospectively on the main outcome, which we discuss in the Data Analysis section. All the subjects were evaluated at the Institute of Osteopathy of Milan ‘S.O.M.A.’ (viale Sarca No. 336-20126, Milan MI). This study was approved by the Institutional Review Board of the Institute of Osteopathy of Milan ‘S.O.M.A’, following the principles of Helsinki for human experimentation. The sample was selected on a series of inclusion criteria, such as the absence of orthodontic therapy in progress or during the previous year, temporomandibular disorders, pain of the oral cavity, mobile protheses, bite, surgery or accidents which occurred in the previous six months, and finally, the presence of at least 24 healthy dental elements. After examining the description of the methods and objectives, the subjects signed an informed consent to proceed with the execution of the dental and osteopathic evaluations, with the analysis through surface electromyography, and finally, with the osteopathic treatment. Furthermore, a consent was signed by each subject for the processing of personal and sensitive data. The 120 subjects suitable for the study were randomly divided into three groups of 40 subjects each, using a random number generator. The sample was divided as follows:

1. Treatment group (12 males, 28 females);
2. Placebo group (17 males, 23 females);
3. Control group (18 males, 22 females).

An extraoral and intraoral clinical examination of the stomatognathic system was carried out on each subject. An orthodontic evaluation was performed to the detect the presence of craniofacial asymmetries, malocclusions following Angle’s classification, discrepancies between centric occlusion (CO) and centric relation (CR), cross-bite, alterations of overjet, and overbite. Then, a functional examination of mandibular movements and Temporomandibular Joint (TMJ) was carried out following the criteria proposed by the Italian Association of Gnathology (AIG), illustrated in Table 1, based on the research from the Diagnostic Criteria for Temporomandibular Disorders (RDC/TMD) Axis I [13].

After the functional examination, at time T0, each patient underwent the first evaluation of the masticatory muscles with sEMG.

The study protocol was divided into three different time groups:

1. T0: the first evaluation with sEMG;
2. T1 (30 min): OMT, placebo treatment, waiting;
3. T2: the second evaluation with sEMG.

### 2.1. Evaluation with Surface Electromyography

Surface electromyography (sEMG) is a noninvasive technique used to analyze the function of the masticatory muscles which, being located in a superficial position, are easily accessible using surface electrodes [14,15]. This exam is painless and innocuous [16].

The sEMG recordings were made using a Teethan wireless system; disposable, pre-gelled, silver/silver chloride bipolar surface electrodes [17], (dimensions 41 mm × 21 mm, diameter 10 mm and interelectrode distance 21 ± 1 mm), were placed on the muscular stomach parallel to the muscle fibers [18], according to the recommendations of sEMG for the noninvasive assessment of muscles (SENIAM) [19]. To standardize the EMG potentials of the analyzed muscles, two 10 mm thick rolls were positioned on the mandibular premolars and molars of each participant, and the 5-s maximum voluntary clenching (MVC) was recorded [20,21]. For the following measurements, the subject was invited to clench as hard as possible for 5 s, maintaining the same level of contraction. The participants sat on a rigid chair, positioned at a distance of 3 m from a smooth wall without visual interferences, with the head unsupported and they were asked to maintain a natural standing position.

Four sets of tests were performed on all subjects at a time T0 and then at a time T2:

1. Sitting with eyes closed in MVC on cotton rolls (COT);
2. Sitting with eyes opened in MVC on cotton rolls;
3. Orthostatic open-eyed position in MVC on cotton rolls;
4. Sitting with eyes closed in MVC (the intercuspal position).

**Figure 1:**
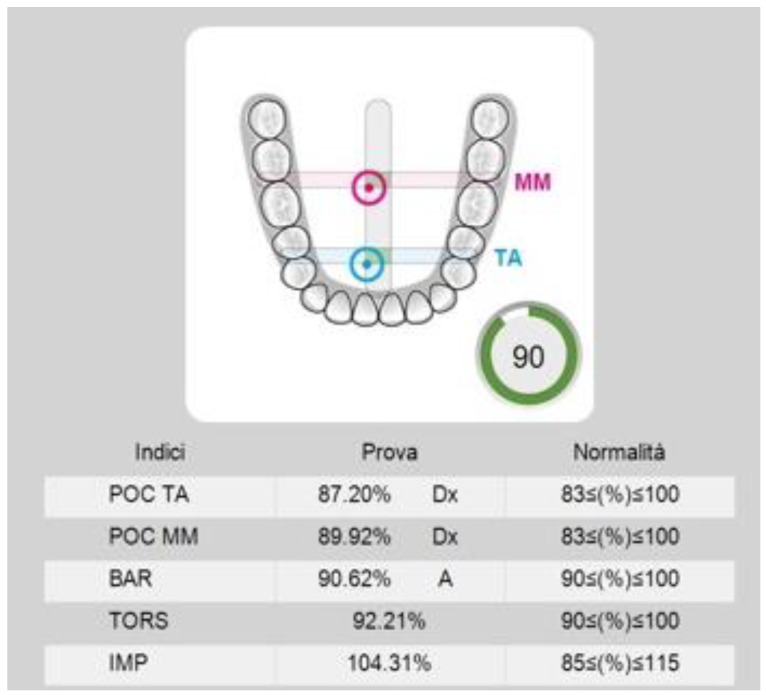
Electromyographic indexes and range of reference. POC TA: percentage overlapping coefficient of right and left Temporalis; POC MM: percentage overlapping coefficient of right and left Masseter muscles; BAR: occlusal barycenter; TORS: Torsion index; IMP: impact index.

The sEMG data analysis was automatically performed by the software. For each participant, the potentials of the analyzed muscles recorded during the MVC tests were expressed as a percentage of the mean potential recorded during the standardization test (MVC on COT) (unit µV/µV × 100) (see Figure 1).

**Figure 2:**
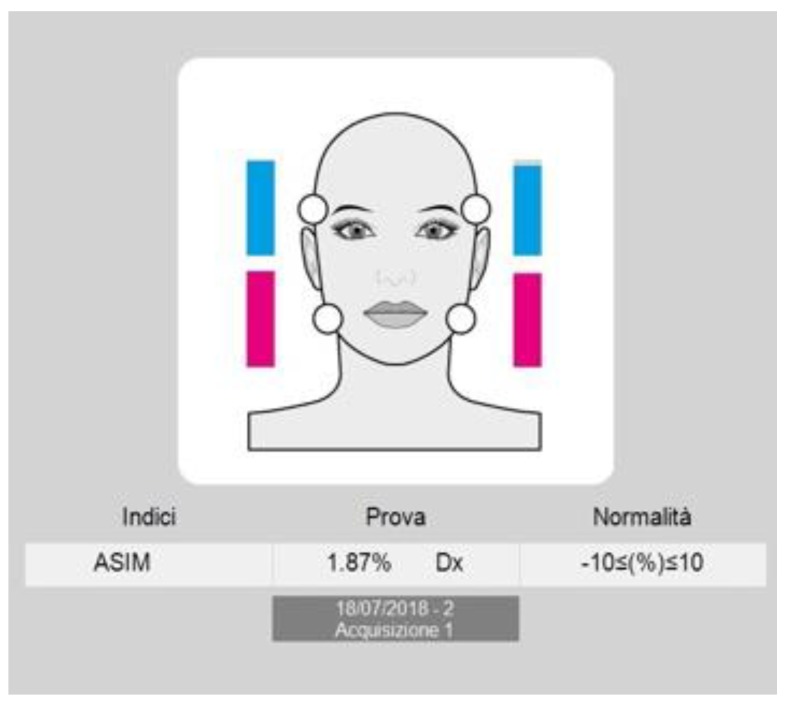
Electromyographic evaluation of muscles asymmetry expressed with the relative ASIM index and through a visual representation of the progressive recruitment of Temporalis muscles in blue and Masseter muscles in Pink.

To assess the muscle symmetry, the EMG waves of paired muscles (right and left masseter and temporalis) were compared by computing the percentage overlapping coefficient (POC). When the muscles contract with perfect symmetry, a POC of 83–100% is obtained. The balance of the contractile activity of contralateral temporalis and masseter muscles (for example, left masseter and right temporalis), expressed by the Torsion index (TORS), ranges between 0% (complete lateral displacing force) and 100% (no lateral displacing force). The Barycenter index (BAR) was considered to investigate if the occlusal barycenter was in an anterior or posterior position. It identifies the most prevalent pair of masticatory muscles as the percentage ratio of the difference between temporalis and masseter standardized potentials (normal range 90–100%). The intensity of muscle work, expressed by the impact index (IMPACT), normally ranges from 100–115%. High values are typical in bruxist patients. Finally, the asymmetry index (ASIM) assessed the symmetry between right and left muscular activity (−10 < (%) > 10) (see Figure 2). The advantage of using these indexes lies in the possibility of evaluating the symmetry, or asymmetry, conditions of muscular activity [22].

### 2.2. Osteopathic Evaluation and OMT-Osteopathic Manipulative Treatment

Each subject underwent an osteopathic evaluation with the aim of observing the posture in static position, performing a dynamic objective examination, and finding areas of tension, mobility restrictions, and greater tissue density.

The subjects from the control group then waited 30 min for the second evaluation and the ones from the placebo group underwent a placebo treatment during the T1 interval.

The subjects of the treatment group also underwent an osteopathic manipulative treatment lasting 30 min.

The treatment included three standardized techniques to be applied to all subjects and three black-box techniques chosen by the operator for the individual subject, based on the previously performed osteopathic assessment.

The standard techniques applied were the direct intraoral and extraoral inhibition of the masseter muscles, direct inhibition of temporalis muscles, and direct inhibition of the external and internal pterygoid muscles.

At time T2, all the subjects were again subjected to surface electromyography.

### 2.3. Data Analysis

An inferential analysis was carried out using the SPSS software version 25 (IBM, Milan, Italy), and R studio version 1.1.463. The values obtained from the evaluations at times T0 and T2 were compared both with the intragroup (OMT, placebo, control) and intergroup (OMT-placebo, OMT-control). The T-test was used in order to compare the averages obtained by studying the three groups and to find out if the differences from T0 and T2 were significant (for a *p*-value ≤ 0.05). Each index has its own reference range which corresponds to an optimal physiological range.

The power of the study was calculated retrospectively on the main outcome, the variable POC TA starting with the sample size (total = 120, 40 in each group), the significance level (α = 0.05), and with three different effect sizes between groups after the intervention (OMT vs. placebo = 0.19; placebo vs. control = 0.42; OMT vs. control = 0.51). The effect size was calculated through Cohen’s D test. In the comparison of OMT with the placebo group, the power was 0.18, for the placebo vs. control group, the power was 0.62, and for the OMT vs. control group, the power was 0.80.

## 3. Results

*OMT intragroup:* in 40% of the subjects, statistically significant changes were found. In the sEMG examinations, the variations detected involved POC TA, POC MM, TORS, and BAR. The average POC TA recorded an improvement in all the measurements, whereas POC MM showed a significant improvement just in the orthostatic position. The average TORS and BAR improved from the T0 to T2 evaluation, although without catching the physiological range identified from the software.

*Placebo intragroup:* the statistically significant changes highlighted in the sEMG evaluations were POC TA and IMPACT, only in 7.5% of the subjects, while the IMPACT index remained within the physiological range, the average POC TA moved away from it, so we can state that there was a worsening in the symmetry of the temporalis muscles activity.

*Control intragroup:* in 17.5% of the measurements, ASIM, TORS, and BAR recorded statistically significant variations remaining in the physiological ranges. 

## 4. Discussion

The results show a statistical significance between the electromyographic indexes measured in T0 and T2.

**Figure 3:**
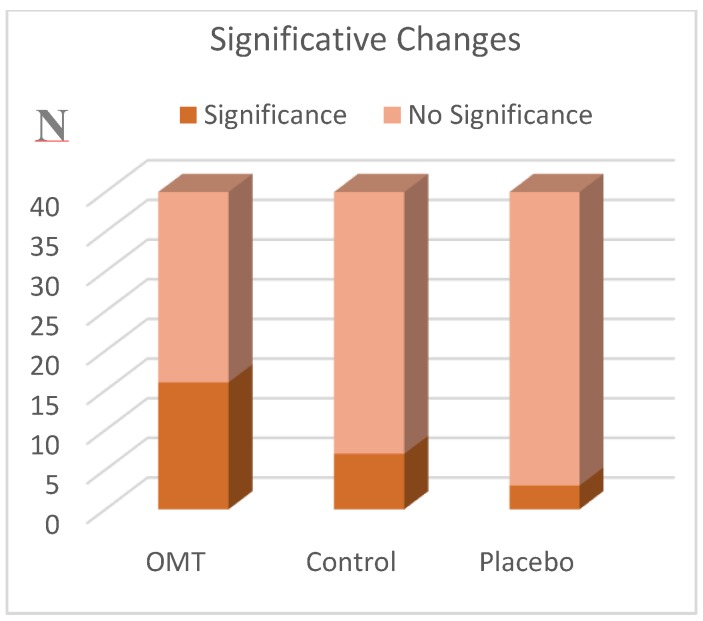
Number of subjects that presented significative variations of their muscular activity between T0 and T2.

The data obtained by analyzing each group showed the greatest number of statistically significant results in the OMT group, as shown in Figure 3; indeed 40% of the subjects of this group improved their muscular activity and no worsening was noted. Several studies show how tissue inhibition reduces pain, increases joint mobility, eliminates adhesions between muscle fibers, improves local circulation, and induces overall relaxation [23].

**Figure 4:**
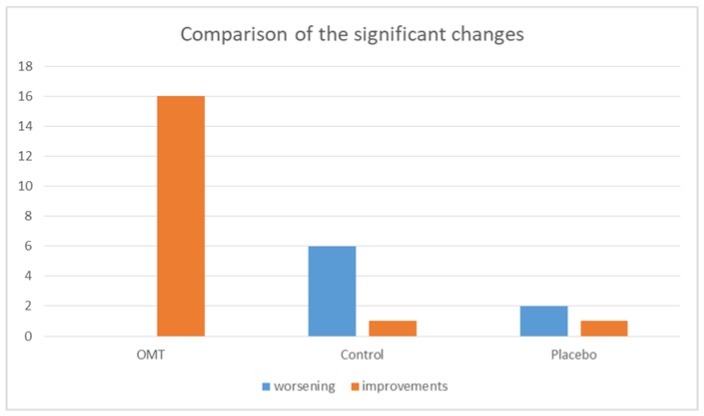
Quality of the significant changes inside each group, considering electromyographic indexes: all the variations in OMT group can be considered improvements whereas the ones in Control and Placebo groups seem more randomly distributed, with a worsening of the absolute value of sEMG indexes.

In the placebo and control groups, we found less statistically significant changes compared to the OMT group. In the placebo group just 7.5% of measurements changed, 75% of which can be considered as a worsening of the sEMG indexes; whereas in the control group, 17.5% of the measurements presented significant changes, 85% of which were considered worsening by the software, as shown in Figure 4.

Analyzing the results obtained by comparing the OMT and placebo groups, there were six statistically significant changes.

Additionally, the comparison between the OMT and control groups showed seven statistically significant changes.

From the intergroup analysis, we can observe that there were more significant changes in the OMT group compared to the placebo and the control groups (Figure 3).

Initial studies have found muscular massage to be effective for persistent back pain [24], and physiotherapy interventions have already been reported in literature as efficacious for patients with TMD pain and restricted motion [25,26].

Given the results of this study, we can consider OMT as a valid aid to improve the muscular activity of the stomatognathic system in preparation for a gnathological intervention. This is in light of the influence that OMT can have on muscular tension and postural compensation. Some of the variations highlighted in the control and placebo groups can be related either to the impossibility of avoiding emotional interferences, and/or to the muscular fatigue that might occur after the first evaluation of patients, and on them having to wait for a second one. The relevance of psychosocial factors indeed has been widely underlined in literature, so much so that psychosocial factors are considered as important for the treatment outcome of TMD, as are initial pain intensity and physical diagnoses [27]. Although this was not our objective, we have to keep in mind the influence of cognitive, behavioral, and emotional aspects on strategies implemented by patients to maintain adequate functioning [28], and that they may also play a role in this case.

What is interesting is the significant minority of variations in the muscular activity of the placebo group. Indeed, despite the lack of a specific therapeutic action, a placebo treatment can elicit real psychobiological responses [29]. Clinically relevant evidence demonstrates that placebo effects can have meaningful therapeutic effects [30], therefore these outcomes should suggest that only a real manipulative treatment can cause more constant, and apparently, positive variations in the electromyographic indexes. Other studies have also shown that complementary and alternative medicine (CAM) treatments, such as massage, spinal manipulation, and mobilization, are significantly more efficacious than placebo treatments in reducing neck and low-back pain after treatment [31].

However, further studies should be conducted on a wider sample to highlight the significance of the differences between placebo and OMT.

In the management of TMDs, the goals of physiotherapeutic regime are to reduce muscular tone, improve kinetic parameters and posture, and decrease risk factors related to the upper quarter by stretching masticatory muscles, increasing TMJ mobility, and influencing muscle strength and proprioception in order to restore normal functioning [32]. In this trial, OMT have shown promising results in influencing muscular activity of the craniofacial region. Furthermore, the osteopath could guide the patient with individualized home exercises. The Delphi study confirmed that there is a consensus among experts that jaw exercises are effective and can be recommended to patients with myalgia in the jaw muscles and restricted mouth opening capacity [33]. Both general dental practitioners and orofacial pain experts could benefit from this interdisciplinary approach for the management of acute and chronic pain.

So far, only a few high quality randomized controlled trials focusing on the therapeutic effectiveness of osteopathic treatment have been published, and most of them failed to prove efficacy. However, the available systematic reviews mainly criticized low methodical quality and paucity of the analyzed osteopathic studies [34].

The main limitations of this study were the absence of a prior effect size and power calculation, the sample size was arbitrarily decided and this, from a retrospective analysis based on one main variable (POC TA), resulted in it only being appropriate for the comparison of OMTs and placebos with controls, but too small to be significant in the comparison between OMTs and placebos. Given the lack of previous studies and the significant differences from the controls, we can only state that manipulative treatments can produce variations in the muscular activity and, starting from this limitation, we are conducting further studies on a wider sample to investigate the differences between placebo treatment and real osteopathic manipulative treatment. Other limitations include the following: the immediate revaluation of the subjects without a period of follow up which should be considered in future studies to evaluate the long-term effect of the treatments; and the choice of evaluating healthy subjects that allows only to analyze the presence of variations in the muscular activity without demonstrating a therapeutic effect of the treatment. Further studies on symptomatic patients should be done in the future to assess the effectiveness of the association between gnathological treatment and osteopathic manipulations.

## 5. Conclusions

The findings of this randomized clinical trial support the effectiveness of osteopathic manipulative treatment on modifying the activity of masticatory muscles. This study demonstrated the positive effect of OMT compared to the placebo treatment.

Our conclusions support the use of OMT in the cranial field, and in particular in dentistry, to achieve muscular balance.

Other studies should be conducted on dysfunctional patients in order to evaluate their symptomatic improvements by associating the gnathological and the osteopathic therapy.

### Clinical Relevance

1. Measurements of the variations of the muscular activity induced by the osteopathic treatment
2. Demonstration of the positive effect of the osteopathic treatment compared to the placebo one
3. Demonstration of the possible usefulness of the osteopathic treatment in dentistry, referred to the muscular balance

## Figures and Tables

**Table 1:** The criteria used to assess the absence of disfunctions.

MANDIBULAR MOVEMENTS	Straight opening pattern without pain Maximum Mouth Opening > 40 mm Lateral and Protrusive movements without pain and restrictions Elastic Assisted Mouth Opening (End-Feel)
TMJ	Absence of noises (clicking, popping, crepitus) Symmetric movements Absence of pain with palpation
